# Rescue and Conservation of Male Adult Alpacas (*Vicugna pacos*) Based on Spermatogonial Stem Cell Biotechnology Using Atomized Black Maca as a Supplement of Cryopreservation Medium

**DOI:** 10.3389/fvets.2021.597964

**Published:** 2021-03-17

**Authors:** Martha Valdivia, Zezé Bravo, Jhakelin Reyes, Gustavo F. Gonzales

**Affiliations:** ^1^Laboratory of Reproductive Physiology, Research Institute “Antonio Raimondi,” Zoology Department, Biological Sciences Faculty, Universidad Nacional Mayor de San Marcos, Lima, Peru; ^2^Endocrine and Reproductive Laboratory, Department of Biological and Physiological Science, and Laboratory of Investigation and Development (LID), Faculty of Sciences and Philosophy, Universidad Peruana Cayetano Heredia, Lima, Peru

**Keywords:** spermatogonia stem cell, alpaca, testicular biopsies, isolated spermatogonial cells, cryopreservation

## Abstract

This is the first time that testicular tissue (*n* = 44) and isolated testicular cells (*n* = 51) were cryopreserved from alpaca testes 24 h postmortem. For this purpose, internally designed freezing media and cryopreservation protocols were used. Testicular tissue fragments (25 mg) and isolated testicular cells were frozen in MTDB (trehalose and black maca), MTD (trehalose), MSDB (sucrose and black maca), and MSD (sucrose) media. Isolated spermatogonial cells were cryopreserved in two ways, before and after proliferation *in vitro*. After cryopreservation, the percentage of cell viability in Group 1 (>50% of cell viability) by trypan blue did not show differences within each group (*p* > 0.05) but showed significant differences when comparing fragments with isolated cells (*p* < 0.05). Spermatogonial stem cells (SSC) were identified by flow cytometry as strong *Dolichos biflorus* agglutinin (sDBA) and mitochondrial activity of SSC as strongly positive for MitoSense (sMitoSense+) in intact mitochondria cells, weakly positive for MitoSense (wMitoSense+) in early apoptosis, and necrosis with 7-Aminoactinomycin-D positive (7-AAD). After freezing, in Group 1M (≥30% sMitoSense+), the fragments did not show differences between the media (*p* > 0.05), but in the isolated cells frozen in MSDB medium, 63.68 ± 8.90% (*p* < 0.05). In Group 2M (<30% sMitoSense+), necrosis (7AAD+) in MSDB medium was 27.03 ± 5.80%, and necrosis in isolated cells was 14.05 ± 9.3% with significant differences between these groups (*p* < 0.05); in sMitoSense+, the isolated cells (34.40 ± 23%) had a higher percentage than the fragments (12.4 ± 5.2) (*p* < 0.05). On the other hand, MSDB and MSD media were significantly higher for isolated cells than for fragments in sDBA+ (*p* < 0.05). On the other hand, the SSC (sDBA+) had significant differences (*p* < 0.05) between fresh cells 7.43 ± 1.3% (sDBA+) compared with those cryopreserved in MSDB medium 1.46 ± 0.34% (sDBA+). Additionally, the proliferated and cryopreserved SSC 6.29 ± 1.17% (sDBA+) did not show significant differences concerning the fresh cells (*p* > 0.05). In conclusion, the black maca showed antioxidant properties when it was included in the freezing medium and, therefore, improved the SSC's conservation of the alpaca. Furthermore, the proliferation of isolated cells *in vitro* produces a higher amount of SSC after thawing them for further preclinical or clinical work.

## Introduction

Spermatogonial stem cells (SSC) are present in minimal amounts in the seminiferous tubules of adult animals; in particular, it is estimated that only 0.03% of cells correspond to SSC (1, 2). SSC have the ability to self-renew, which guarantees balanced male fertility (3). This property makes them attractive as an advanced biotechnological tool for conserving genetic material from wildlife and elite animals, since the establishment of SSC banks (4).

In addition, testicular tissue preserved *ex vivo* through the freezing of testicular biopsies or isolated SSC from adult individuals could serve as a reservoir for the rescue and conservation of male fertility (4). In fact, the preservation of SSC allows for the rescue of important genetic material. Therefore, these cells can help to preserve male fertility in individuals from childhood to adulthood and in animals with good reproductive characteristics. Indeed, frozen and thawed testis tissues or isolated cells can be transplanted to the same individual from which the progenitor tissues were derived or to an individual of a lesser race, making the recipient individual produce male animal gametes from these sources (5).

Moreover, SSC cryopreservation would allow the study, rescue, and conservation of SSC of animals with high genetic value (6), including animals with a high economic impact in Peru, such as alpacas, and can thus be used for reproductive genetic management of Peruvian alpacas producing good fiber. In animal models, it has been described that frozen testis tissue can undergo differentiation after cryopreservation, thereby producing primary spermatocytes that eventually differentiate into round spermatocytes and ensuring the production of spermatozoa (1, 7, 8). Therefore, cryopreservation of testicular tissue shows excellent potential in assisting male fertility (9, 10) because spermatozoa and SSC can be rescued from testicular biopsies after thawing and be used for assisted reproduction techniques of high complexity, such as intracytoplasmic insemination (ICSI), with low abortion rates (11, 12).

Currently, several research groups are committed in developing biotechnologies in the fields of both isolation, cryopreservation, and transplantation of SSC, highlighting the possible applications of SSC (13). For example, extensive work has been carried out on humans (11), mice (14, 15), cattle (16), pigs (17), and alpacas (18), where it was possible to identify SSC as well as early differentiating SSC using molecular markers and *Dolichos biflorus* agglutinin (DBA) (19).

Cryopreservation of isolated SSC or SSC in the form of testicular biopsies has the potential, in the long term, to support highly efficient methods of reproductive biotechnology for conserving male genetic material and could lay the foundation for the creation of SSC banks for the Peruvian alpaca, generating potentially useful new alternatives to conserve male fertility in alpaca elites. Alpaca is one of the leading livestock species of the high Andean region in Peru, with great economic impact. Peru hosts more than 87% of the worldwide alpaca population. Distributed in a more significant proportion in Puno, Cuzco, and Huancavelica, 85% of the alpaca population corresponds to the Huacaya breed (with 95% white and 5% colored) and 15% to the Suri breed (7), whose productivity is currently declining. Peruvian alpacas have high genetic variability (20, 21); however, fiber productivity decreases annually by 2.3%.

Various research groups have started to study and characterize this species from the reproductive perspective, with genetic improvement as one of the main objectives (22, 23). Advances have been made in the cryopreservation of semen and epididymal sperm, artificial insemination, and embryo culture as well as in the evaluation of genetic diversity, in order to establish strategies for the selection of reproducers and the application of appropriate technologies for this species (24). However, alpaca reproduction still shows considerable difficulties, such as a long gestation time, difficult handling, and establishing artificial insemination programs. Indeed, this method is limited by semen collection or by low post-thawing motility of epididymal sperm (25). Unfortunately, adequate protocols for semen preservation, one of the most widely used techniques in reproductive biotechnology in other domestic species, have not yet been established for alpacas; therefore, it is necessary to study germ cells from different perspectives.

In adult alpacas, the presence of SSC was identified using molecular markers (Zbtb16, Integrin β1) and by flow cytometry (FC) after enzymatic isolation from testicles of adult alpacas and staining with DBA, as SSC are strongly DBA-positive (sDBA+) cells (18) that can proliferate and differentiate *in vitro* (26). Because of this property, freezing of gonadal tissue or isolated spermatogonial cells can facilitate the conservation of SSC as well as testicular sperm, which could be used in several reproductive procedures (6, 27, 28).

Unfortunately, it was never possible to freeze testicular biopsies or testicular fragments of alpaca with traditional cryopreservation media; however, in some species, such as mice, pigs, cattle, and humans, some freezing media and techniques have been optimized. For instance, vitrification-based freezing of human testicular biopsies has been described, with promising results for the cryopreservation of testicular tissue (9). Moreover, it has been observed that freezing testicular tissue of mice with dimethylsulfoxide (DMSO) and propanediol (PROH) preserves the ability of testicular sperm to produce embryos *via* ICSI, which, when transferred to pseudopregnant females, generate new individuals (10).

Therefore, supplementation of the medium with different additives is a strategy to improve the efficiency of cryopreservation. For instance, the use of sugars, such as trehalose and sucrose, as non-permeable cryoprotectants is recommended to increase the recovery, viability, proliferative capacity, and colonization efficiency of undifferentiated spermatogonia after thawing in species, such as mice (15), pigs (17, 29), and cattle (16). In addition, cryopreservation media have also been supplemented with additives, such as collagen, laminin, and antioxidants. The latter can increase the survival of cells by scavenging the reactive oxygen species generated during the cryopreservation process, which can damage cellular structures, such as membranes or proteins, thereby the sensitivity to freeze/thaw processes, or DNA integrity, thus reducing the viability and proliferative capacity of SSC (13).

Indeed, there is an obvious need to preserve male fertility in individuals from childhood to adulthood (for example in oncology patients) and animals with some good reproductive characteristics that become evident during the adult stage. Therefore, conserving testicular tissue *ex vivo* through the freezing of testicular biopsies or isolated SSC of adult individuals could serve as a reservoir for the rescue and conservation of male fertility.

Nevertheless, alpaca sperm cryopreservation has not been used extensively because of its limiting step of semen collection. In addition, alpaca epididymal sperm presents low sperm motility after thawing, so it is impossible to use it in artificial insemination programs. On the other hand, freezing of gonadal tissue or isolated spermatogonial cells would facilitate the conservation of SSC and spermatozoa and contribute to the preservation fertility of alpacas.

Therefore, SSC are attractive as an advanced biotechnological tool for the conservation of male genetic material of wildlife and elite animals. SSC freezing/thawing would allow the study, rescue, and conservation of SSC of animals with high genetic value, including animals with a high economic impact in Peru, such as alpacas, and thus can be used for reproductive genetic management in Peruvian alpacas producing good fiber. Furthermore, in animal models, it has been described that differentiation of SSC ensures spermatozoa production (1, 7, 8). In adult alpacas, the presence of SSC has been identified using molecular markers (Zbtb16, Integrin β1) and by FC after enzymatic isolation from testicles of adult alpacas and staining with DBA, as SSC are sDBA+ cells [mean ± standard deviation (SD) of sDBA+] corresponding to SSC = 4.43 ± 0.68% (18), which can proliferate and differentiate *in vitro* (26, 30). Cryopreservation of testicular tissue shows promising applications in male fertility (9, 10). Indeed, testicular biopsies, spermatozoa, and SSC can be recovered after thawing and used in assisted reproduction techniques of high complexity, such as ICSI, with low abortion rates (12).

It has been observed that administering maca *in vivo* to mice with physically induced subfertility during 35-days treatments reduces sperm DNA fragmentation, from 11.1 ± 19.29 to 2.29 ± 2.30% (mean ± SD) while increasing sperm concentration and mobility (31). In addition, different biological responses to various ecotypes of maca have been observed (32). For example, maca can exert anti-stress energizing effects and scavenge free radicals and provide cytoprotection under oxidative stress conditions (33–35). Unfortunately, it was never possible to freeze testicular biopsies or testicular fragments of alpaca with traditional cryopreservation media.

After thawing human testes tissue, the testicular sperm can be used for ICSI with low abortion rates (12). Advances in tissue cryopreservation, as well as in germ cell transplantation and testicular biopsy technology, open new horizons for the preservation of male fertility (36, 37), offering alternatives to cancer patients affected in their fertility since childhood (38). Indeed, cryopreservation of testis biopsies can help children who are subjected to aggressive treatments preserve future fertility after transplant (39, 40).

Maca (*Lepidium meyenii* Walp), a plant of the Brassicaceae family from the high Andean region of Peru, grows at 4,000 m.a.s.l., whose traditional use has been for 2,000 years as food and also has a medicinal role (41). Its consumption has been shown to improve fatigue (42), spermatogenesis (32, 41), and erectile dysfunction in animals (43) and in humans (44). Different biological responses of maca ecotypes (32) provide energizing anti-stress and antioxidant effects (33, 34, 45), natural cytoprotection (46), and cytoprotection in oxidative stress conditions (35). Through nuclear magnetic resonance (NMR) and biochemical analysis, Zhao et al. identified that in maca, there are proteins, lipids, carbohydrates, and unsaturated fatty acids and minerals (47). Maca shows properties as a natural potential cryoprotection agent (CPA) to alpaca SSC. On the other hand, it has been observed that maca reduces sperm DNA fragmentation in animals with chemically induced subfertility (31); moreover, long-term treatment with black maca has been reported to enhance daily sperm production and increase sperm motility. These findings indicate differences in the biological response to various maca ecotypes (32), such as anti-stress energizing effects, free radical scavenging, and cytoprotection under oxidative stress conditions (33–35).

For this purpose, we developed cryopreservation media with permeable and non-permeable cryoprotective agents and the addition of a natural supplement, black maca, whose components exert antioxidant effects in mice (31). It was never possible to freeze testicular biopsies or testicular fragments of alpaca with traditional cryopreservation media; however, in some species, such as mice, pigs, cattle, and humans, freezing of testicular material has been successfully performed.

We propose that our freezing media would help tissue and cell cryogenics for promising applications in prepubertal patients and even adult men, as well as for elite animals of high value and genetic lineage, and economic impact, such as alpacas, valued for their quality of fiber and meat, and the conservation of wildlife animals.

Here, we aimed to evaluate the cytoprotective effect of black maca during cryopreservation in both testicular biopsies and isolated SSC before and after *in vitro* proliferation by analyzing mitochondrial activity, early apoptosis, and post-thawing necrosis.

## Materials and Methods

### Animals

Fifty-one adult male alpacas (4–6 years old), coming from family breeding facilities of the populated centers of the Lacchoc, Cachimayo, Huaracco, and Carhuancho areas (Huancavelica, Peru), were slaughtered in the Municipal Camal of Chuñuranra town, at 3,680 m.a.s.l. The environmental temperature of the area fluctuates between 6 and 8°C, with an average annual rainfall between 400 and 700 mm and an altitude between 3,000 and 4,600 m.a.s.l. Testicles with epididymis were recovered immediately after slaughter in 0.9% NaCl and transported in isothermal boxes provided with gel ice to maintain the cold chain during transport (24 h) to the Laboratory of Reproductive Physiology, Faculty of Biological Sciences, Universidad Nacional Mayor de San Marcos (UNMSM), Lima, Peru.

### Samples

Testicles and epididymis were washed in phosphate-buffered saline with 0.1 mg/ml penicillin, 0.1 mg/ml streptomycin, and 0.5 mg/ml gentamicin soon after arrival at the laboratory (UNMSM) in Lima, Peru. The tunica albuginea tissue was removed in sterile conditions. Testes that weighed ≥8 g and exhibited progressive epididymal sperm motility of ≥30% were selected (26).

Epididymal sperm parameters were evaluated according to Organization WH (48). Sperm motility was evaluated and classified as follows: P, progressive motility; NP, non-progressive motility; and I, immotile. Several microscopic fields were evaluated using a Scientific i4 Series light microscope (LW Scientific, Lawrenceville, GA, USA) and a video recorder with an OmniVID camera, and the MicroCap v3.0 software (LW) values were expressed as percentages, based on the observation of 100 spermatozoa in each sample. Samples were classified as good (≥30% of progressive motility of epididymal spermatozoa), regular (20–29% of progressive motility of epididymal spermatozoa), and poor (from 0 to 19% of progressive motility of epididymal spermatozoa), as described by Valdivia et al. (18). Only samples exhibiting good quality were used for the thawing protocol of fragments and isolated spermatogonial cells. Sperm concentration was determined using a hemocytometer and expressed in millions of spermatozoa/ml.

Forty-four samples were frozen as fragments and 32 samples as isolated spermatogonial cells. Eight samples frozen as fragments and isolated spermatogonial cells were analyzed by FC. Fifteen additional samples were used for the *in vitro* proliferation experiment.

Freezing media for fragments and isolated spermatogonial cells were supplemented with atomized black maca (Juvens^®^ Cayenatur, Lima, Peru) as described by the Research Circle of Plants with Effect on Health (grant no. 010-2014-FONDECYT). Botanical samples were deposited in the HEPLAME MG-2015 (Herbarium of Medicinal Plants, Section of Pharmaceutical Sciences, Faculty of Sciences and Philosophy, Universidad Peruana Cayetano Heredia). Maca components have been previously characterized by NMR (49).

### Recovery of Testicular Tissue Fragments and Cryopreservation (Protocol LFR-UNMSM-2)

Longitudinal cuts of testicles were made, and three 25-mg fragments of tissue (8 × 2 × 2 mm) were obtained from each sample and immediately transferred to 80 μl of four media at room temperature: MTDB (HAM-F10 modified, 0.2 M of trehalose, 10% DMSO, and 20 mg/ml atomized black maca Juvens^®^), MTD (HAM-F10 modified, 0.2 M of trehalose, and 10% DMSO), MSDB (HAM-F10 modified, 0.2 M of sucrose, 10% DMSO, and 20 mg/ml atomized black maca Juvens^®^), or MSD (HAM-F10 modified, 0.2 M of sucrose, and 10% DMSO).

Samples (*n* = 44) were placed for stabilization for 1 h in a water bath at 4°C. Subsequently, cryopreservation was performed in the Freeze Control CL 3300 heat-controlled system (CryoLogic, Victoria, Australia) using the Cryogenesis IV software. Our in-house designed freezing protocol of testicular fragments is called protocol LFR-UNMSM-2 (30). Freezing begins at 4°C and ends at −35°C. After the process, the samples were suspended in nitrogen vapors (−80°C) for 12 h. Then, the samples were immersed in liquid nitrogen at −196°C. Samples were slowly thawed from −196°C, placing them in a 12 × 8 cm polystyrene box uncovered in room temperature at 19°C for 5 min then to 37°C until it thaws completely (5 min more approximately).

### Isolation of SSC

Testicular tissue (0.8 g), except rete testis, was cut into small pieces in Minimum Essential Medium (MEM) supplemented with 100 μg/ml penicillin and 100 μg/ml streptomycin; then, fragments were filtered through a metal mesh in order to disaggregate the tissue into spermatogonial cell suspensions. Subsequently, the samples were processed according to Izadyar et al. (50), by two steps of enzymatic digestion (ED): ED1 and ED2. ED1 was performed in MEM supplemented with 1 mg/ml collagenase, 26.3 μl/ml DNase, and 0.5 mg/ml hyaluronidase for 30 min at 32°C in continuous motion, and then the suspension was washed with MEM by centrifugation and resuspension. The cells were then suspended in ED2 medium, which was supplemented with 1 mg/ml collagenase and 0.5 mg/ml hyaluronidase according to the previously described methodology (50). The cell suspension was centrifuged at 1,800 rpm (300 × *g*) for 5 min, the supernatant was discarded, and the cell pellet was washed three times in MEM supplemented with penicillin and streptomycin. Finally, cell viability was analyzed *via* trypan blue exclusion assay. In this assay, non-stained cells were considered alive, whereas cells staining blue were dead. Samples (*n* = 32) showing ≥80% of cell viability by trypan blue assay were used for cryopreservation and *in vitro* culture experiments.

### Cryopreservation of Isolated Spermatogonial Cells (Protocol LFR-UNMSM-3)

Similar to testicular fragments, 2 × 10^6^/ml isolated spermatogonial cells (*n* = 32) were mixed with MTDB, MTD, MSDB, or MSD medium at 1:1 ratio in a total volume of 80 μl. Next, 40 μl of the cell suspension was added to 40 μl of four cryopreservation media: MTDB (HAM-F10 modified, 0.2 M of trehalose, 10% DMSO, and 20 mg/ml atomized black maca Juvens^®^), MTD (HAM-F10 modified, 0.2 M of trehalose, and 10% DMSO), MSDB (HAM-F10 modified, 0.2 M of sucrose, 10% DMSO, and 20 mg/ml atomized black maca Juvens^®^), or MSD (HAM-F10 modified, 0.2 M of sucrose, and 10% DMSO) at room temperature. After stabilization (for 1 h at 4°C), the samples were frozen in the heat-controlled system using the Cryogenesis IV software and our in-house designed protocol for cell suspensions (LFR-UNMSM-3) (30). The temperature decrease continued at a rate of 2°C/min until reaching a temperature of −7°C, set to last 11 min. At this temperature, ice sowing was induced by seeding. Subsequently, at a rate of 0.3°C/min, the temperature reached −30°C and was then immediately brought down to −84°C at a rate of 6°C/min. At the end of the freezing process, the samples were withdrawn from the cryochamber, suspended in liquid nitrogen vapors for 12 h, and then immersed in liquid nitrogen at a temperature of −196°C. Samples were thawed from −196 to 18°C for 5 min and incubated at 37°C.

### *In vitro* Culture of Isolated Spermatogonial Cells

Other aliquots of the spermatogonial cell suspensions (1 × 10^6^ cells/ml) were cultured *in vitro* according to Valdivia et al. (26) in polystyrene culture plates (SPL Life Science) with Dulbecco's Modified Eagle Medium (DMEM; Gibco) supplemented with 0.29 mg/ml l-glutamine (Sigma-Aldrich), 10 μl/ml non-essential amino acids (Sigma-Aldrich), 100 μg/ml penicillin (Sigma-Aldrich), 100 μg/ml streptomycin (Sigma-Aldrich), 1 μl/ml insulin–selenite–transferrin sodium (Sigma-Aldrich), 30 mg/ml pyruvic acid (Sigma-Aldrich), 1 μl/ml lactic acid (Sigma-Aldrich), 0.5% bovine serum albumin (Sigma-Aldrich), 1% fetal bovine serum (Sigma-Aldrich), and 1% human milk (donated by an anonymous mother). Cells were cultured at 37°C and 5% CO_2_ for 15 days. After that, the cells were cryopreserved and thawed following the protocol LFR-UNMSM-03 as described above.

### Quantification of SSC and Cells Displaying Normal Mitochondrial Membrane Potential After Cryopreservation by FC

Similarly, the amount of SSC was evaluated in 1 × 10^6^ cells/ml per sample, from fragments (*n* = 8) and isolated cells (*n* = 8) post-thawing, after labeling with 100 μg/ml of the fluorescein isothiocyanate-conjugated DBA. Through this method, we identified SSC as sDBA+ cells, early differentiating cells as weakly marked (wDBA+) cells, and differentiated round cells as DBA-negative (DBA–) cells, according to Valdivia et al. (26). The mitochondrial state of the post-thaw cells was determined with the FlowCellect MitoPotential Red Kit (Merck), which is a dual parameter assay kit for FC including MitoSense Red, a fluorescent cationic dye, and the DNA intercalator 7-Aminoact (7AAD). We classified cells as follows: (1) strongly MitoSense-positive (sMitoSense+) cells with intact mitochondrial activity; weakly MitoSense-positive (wMitoSense+) cells in early apoptosis; and (2) MitoSense-negative (MitoSense–) cells, not labeled by MitoSense Red but showing an intense orange fluorescence due to 7AAD. Cell population analysis was performed with the Amnis imaging flow cytometer (Merck). Histograms produced for each fluorochrome permit with bin tool to calculate the amount of the cell population according to the intensity of the fluorochromes selected. A total of 10,000 events were recorded for analysis using the IDEAS software (EMD Millipore, Burlington, MA, USA). Unfrozen spermatogonial cells, proliferated cells, and fresh cells exposed to UV light were used as control for MitoSense, DBA, and 7AAD staining, respectively.

### Statistical Analysis

Differences between media with or without black maca Juvens^®^ were tested with Kruskal–Wallis tests (*p* < 0.05). Likewise, comparative tests were performed to highlight significant differences between frozen sample types (fragments vs. cells) using the Mann–Whitney *U* test. Statistical analysis was performed with the SPSS statistical package. Tests of homogeneity (Levene's test) and normality (Shapiro–Wilk test) were also carried out.

### Ethical Aspects

This study was evaluated, registered, and approved by the UPCH Institutional Ethics Committee for the Use of Animals (CODE SIDISI No. 0000066755). There is no special interest; there is no bias or particular interest in the formulation of the media, the natural supplements used, or the protocols.

## Results

To develop an effective alpaca SSC freezing protocol, we collected testicles from 51 animals after 24 h from death. We selected only samples scoring above a minimal threshold for the following parameters on testicular weight, round spermatogonial cell concentration and viability, and sperm viability and motility (Table 1). These samples were included in the study to optimize the freezing/thawing process of fragments and isolated SSC. Our results were highly variable, grouped according to spermatogonial cell viability with trypan blue dye: Group 1: >50%, Group 2: 30–50%, and Group 3: <30%. Mitochondrial activity status was identified in spermatogonial cells using MitoSense fluorochrome and grouped according to mitochondrial activity as Group 1M (≥30% sMitoSense+) and Group 2M (<30% sMitoSense).

**Table 1:** Samples characterization by parameter ranges.

	**Range**
Number of samples (*n*)	55
Testicular weight (g)	8–19.94
Round spermatogonial cell concentration (millions)	20–171.5
Round spermatogonial viability (%)	80–97.4
Sperm concentration/epididyme (millions/mL)	9–1,029
Sperm viability (%)	30–93.9

### Evaluation of Cell Viability by Trypan Blue Staining After the Freeze/Thaw Process

After performing the thawing process, the viability of single round cells, derived from fragments by ED as described for isolated cells, and of isolated frozen cells was evaluated after storage at −196°C for 1–166 days and 1–148 days, respectively, by trypan blue exclusion assay. Forty-four fragment and 32 isolated spermatogonial cell samples were used (Table 2). The 37 fragment samples were classified into three groups according to the percentage of viability in MTDB, MTD, MSDB, and MSD media: Group 1, >50%; Group 2, 30–50%; and Group 3, <30%. Group 1 included samples with the highest percentage of viability. In particular, cell viability from thawed fragments belonging to Group 1 was 78.51 ± 2.91% in 60% of evaluated samples in MTDB medium (*n* = 23), 72.96 ± 2.80% in 60% of evaluated samples in MTD medium (*n* = 23), 72.32 ± 2.39% in 64% of evaluated samples in MSDB medium (*n* = 23), and 74.42 ± 2.46% in 60% of evaluated samples in MSD medium (*n* = 23, Table 2). The mean percentage of viability of fragment-derived cells did not significantly differ (*p* > 0.05) among the four freezing media, not even among Groups 1, 2, and 3.

**Table 2:** Mean Percentage of cell Viability calculated by Trypan blue exclusion assay.

	$\bar{\text{x}}$ **±** **SE (*****n*****)**			
	**MTDB**	**MTD**	**MSDB**	**MSD**
**Fragments (37)**				
Group 1: > 50%	78.51 ± 2.91 (23)^a^	72.96 ± 2.80 (23)^a^	72.32 ± 2.39 (23)^a^	74.42 ± 2.46 (23)^a^
Group 2: 30-50%	40.90 ± 2.13 (09)^a^	38.55 ± 2.33 (09)^a^	41.70 ± 1.55 (09)^a^	44.71 ± 1.28 (09)^a^
Group 3: <30%	19.20 ± 4.66 (05) ^a^	17.03 ± 6.72 (05)^a^	19.14 ±4.45 (05)^a^	19.20 ± 4.66 (05)^a^
**Isolated cells (32)**				
Group 1: > 50%	63.60 ± 3.35 (09)^b^	61.75 ± 1.57 (12)^b^	60.51 ± 1.87(8)^b^	59.31 ±1.84 (12)^b^
Group 2: 30–50%	39.65 ± 1.52 (11)^a^	36.42 ± 1.67 (11)^a^	40.59± 1.63 (15)^a^	39.6 2± 1.93 (11)^a^
Group 3: <30%	20.88 ± 2.12 (12)^a^	20.33 ± 2.23(09)^a^	12.79 ±2.68 (09)^a^	19.92 ± 2.08 (09)^a^

a,b *Different letters show statistical differences*.

Similarly, 32 samples of isolated spermatogonial cells were thawed, and cell viability was evaluated by trypan blue staining. Group 1 included samples with the highest percentage of viability. In particular, cell viability from thawed isolated spermatogonial cells was 63.60 ± 3.35% in 28% of evaluated samples in MTDB medium (*n* = 9), 61.75 ± 1.57% in 37.5% of evaluated samples in MTD medium (*n* = 12), 60.5 ± 1.87 in 25% of evaluated samples in MSDB medium (*n* = 8), and 59.31 ± 1.84% in 37.5% of evaluated samples in MSD medium (*n* = 12). There were no significant differences in cell viability between cells frozen in different media nor among Groups 1, 2, and 3 (Table 2). However, when compared, the viability in cell derived from fragments vs. isolated cell of Group 1 did significantly differ (*p* < 0.05) in all media (Table 2).

### Quantification of SSC and Determination of Mitochondrial Membrane Potential After Cryopreservation by FC

The percentage of SSC (sDBA+) and their mitochondrial membrane potential were analyzed in cell extracts from thawed fragments and isolated spermatogonia.

Samples were classified according to the intensity of MitoSense staining into two groups: Group 1M, ≥30% of sMitoSense+ cells (Table 3) and Group 2M, <30% of sMitoSense+ cells, for identifying the best freezing medium with good and low samples, respectively (Table 4).

**Table 3:** Mean percentage of spermatogonial stem cell with intact mitochondrial activity (sMitoSense+), early apoptosis (wMitoSense) and necrosis (7AAD+) in-group 1M.

**Group 1M: ≥30% (sMitoSense+)**	$\bar{\text{x}}$ **±** **S.E(%)**			
**TESTICULAR FRAGMENT (*n* = 8)**	**MTDB**	**MTD**	**MSDB**	**MSD**
sDBA+	8.43 ± 3.37^a^	13.52 ± 1.27^a^	9.67 ± 2.22^a^	7.41 ± 2.37^a^
wDBA+	70.3 ± 6.48^a^	68.73 ± 2.32^a^	71.98 ± 1.60^a^	67.58 ± 2.88^a^
DBA-	11.15 ± 5.38^a^	15.40 ± 8.65^a^	15.92 ± 4.15^a^	14.29 ± 9.78^a^
sMitoSense+	57.20 ± 9.57^a^	34.07 ± 12.20^a^	48.53 ± 10.46^a^	41.96 ± 12.92^a^
wMitoSense+	23.7 ± 6.25^a^	29.57 ± 19.07^a^	20.55 ± 5.41^a^	34.18 ± 5.88^a^
7AAD+	11.15 ± 5.38^a^	15.40 ± 8.65^a^	16.00 ± 4.15^a^	14.29 ± 9.78^a^
**ISOLATED SPERMATOGONIAL (*****n*** **=** **8)**				
sDBA+	5.95 ± 2.62^a^	5.07 ± 1.87^a^	2.73 ± 2.02^b^	1.09 ± 0.84^b^
wDBA+	34.50 ± 7.57^b^	40.92 ± 6.20^a^	39.05 ± 13.56^a^	41.17 ± 6.94^a^
DBA-	53.97 ± 9.95^a^	47.42 ± 3.28^a^	51.45 ± 13.80^a^	52.02 ± 6.07^a^
sMitoSense+	46.90 ± 2.73^a^	43.58 ± 2.95^a^	63.68 ± 8.90^b^	53.00 ± 11.65^a^
wMitoSense+	25.58 ± 4.88^a^	31.65 ± 4.28^a^	32.46 ± 12.05^a^	33.23 ± 5.57^a^
7AAD+	6.71 ± 2.29^a^	8.10 ± 2.41^a^	5.18 ± 2.13^a^	10.03 ± 3.70^a^

a,b *Different letters show statistical differences*.

**Table 4:** Mean percentage of spermatogonial stem cells with intact mitochondrial membranes (sMitoSense+), undergoing, early apoptosis (wMitoSense+) and necrosis (7AAD+) in group 2M.

**Group 2M: <30% (sMitosense+)**	$\bar{\text{x}}$ **±** **EE**			
**TESTICULAR FRAGMENT (*n =* 8)**	**MTDB**	**MTD**	**MSDB**	**MSD**
sDBA+	2.96 ± 2.54^a^	2.68 ± 2.68^a^	2.96 ± 2.55^a^	2.68 ± 2.69^a^
wDBA+	54.87 ± 16.43^a^	48.65 ± 22.11^a^	57.6 ± 19.33^a^	65.8 ± 18.15^a^
DBA-	39.69 ± 17.72^a^	47.45 ± 24.20^a^	38.4 ± 19.76^a^	25.80 ± 20.05^a^
sMitoSense+	16.50 ± 11.27^a^	20.92 ± 12.42^a^	12.41 ± 5.19^a^	16.64 ± 8.13^a^
wMitoSense+	57.85 ± 14.28^a^	58.9 ± 12.26^a^	57.23 ± 6.73^a^	50.90 ± 14.77^a^
7AAD+	19.76 ± 7.45^a^	18.52 ± 4.36^a^	27.03 ± 5.80^b^	13.78 ± 0.03^a^
**ISOLATED SPERMATOGONIAL** (*n =* 8)				
sDBA+	21.9 ± 11.40^b^	6.8 ± 6.8^a^	3.36 ± 1.52^a^	5.51 ± 1.69^a^
wDBA+	43.18 ± 32.02^a^	29.62 ± 20.49^b^	53.00 ± 10.60^a^	66.75 ± 12.25^a^
DBA-	28.7 ± 17.90^a^	61.1 ± 28.30^b^	39.15 ± 7.15^a^	23.9 ± 9.90^a^
sMitoSense+	2.05 ± 1.51^a^	9.6 ± 5.90^a^	34.40 ± 23^b^	27.7 ± 8.70^b^
wMitoSense+	65.1 ± 16.8^a^	52.2 ± 6.40^a^	39.45 ± 16.95^a^	43.80 ± 0.10^a^
7AAD+	23.04 ± 13.36^a^	24.05 ± 12.55^a^	14.51 ± 9.29^a^	16.65 ± 11.35^a^

a,b *Different letters show statistical differences*.

**Figure 1:**
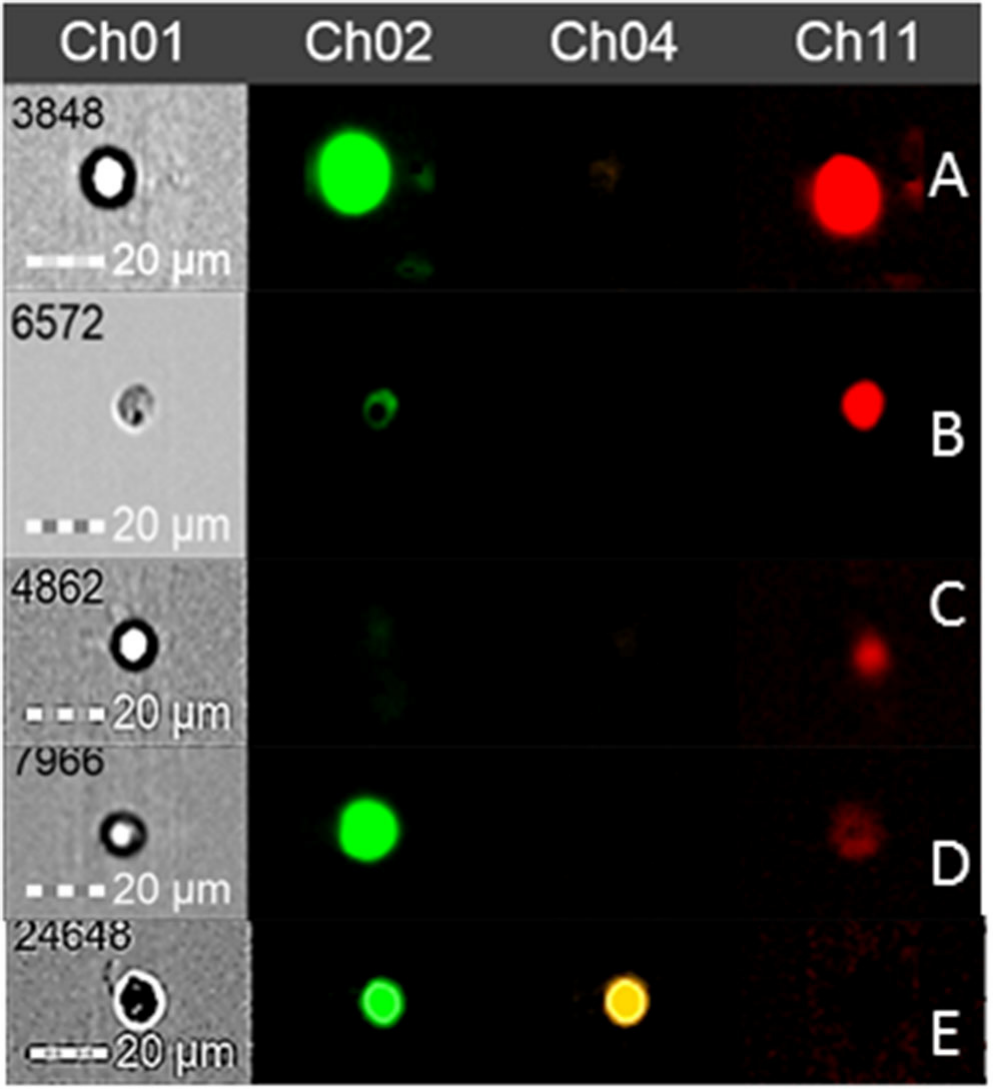
Round spermatogonial cells observed by flow cytometry with DBA (Channel 2), 7AAD (Channel 4), and MitoSense (Channel 11) fluorochromes. **(A)** SSC (sDBA+) with intact mitochondrial membrane (sMitoSense+). **(B)** Early differentiating SSC (wDBA+) with intact mitochondrial membrane (sMitoSense+). **(C)** Differentiated round spermatogonial cell (DBA-) in early apoptosis (wMitoSense+). **(D)** SSC (sDBA+) in early apoptosis (wMitoSense+). **(E)** Necrotic SSC (7AAD+).

Further, cells displaying strong mitochondrial activity were revealed as sMitoSense+ cells, cells undergoing early apoptosis as wMitoSense+ cells, and necrotic cells as 7AAD+ cells (Figure 1). In Group 1M, the percentage of SSC (sDBA+ cells) and sMitoSense+ for SSC derived from fragments among MTDB, MTD, MSDB, and MSD freezing media did not significantly differ (*p* > 0.05). On the other hand, we found statistically significant differences (*p* < 0.05) in sDBA+% in MSDB (2.73 ± 2.02%) and MSD (1.09 ± 0.84%) with MTDB (5.95 ± 2.62%) and MTD (5.07 ± 1.87%) freezing media in isolated cryopreserved cell and with cell derived from fragments cryopreserved in MSDB (9.67 ± 2.22%) and MSD (7.41 ± 2.37%) media (Table 3).

**Figure 2:**
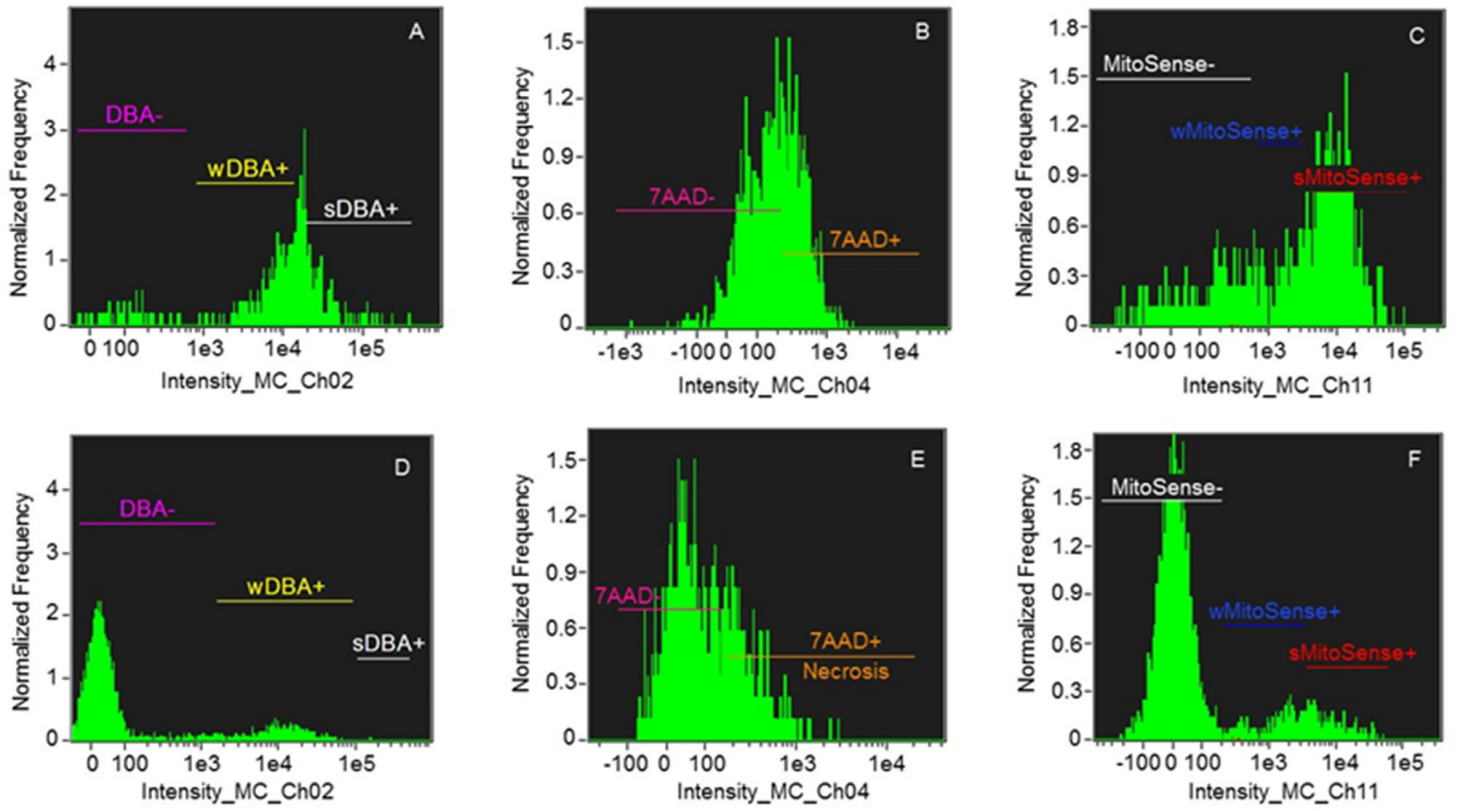
Histograms of samples of Group 1: > 30% sMitoSense+ **(A–C)** and Group 2 < 30% sMitoSense+ **(D–F)**. **(A,D)** DBA histograms showing strongly DBA-positive (sDBA+) cells corresponding to SSC, weakly DBA-positive (wDBA+) cells corresponding to early differentiating SSC, and DBA-negative (DBA-) cells corresponding to differentiated round spermatogonia. **(B,E)** Histograms of necrotic cells (7AAD+). **(C,F)** Histograms of cells with intact mitochondrial membranes (sMitoSense+), weakly MitoSense-positive (wMitoSense+) cells, undergoing early apoptosis, and MitoSense-negative (MitoSense−) cells.

In particular, the high percentage of sMitoSense+ cells among thawed isolated spermatogonia was 63.68 ± 8.90% (Figure 2, Table 3) and significantly higher than MTDB, MTD, and MSD media and with cell derived from fragments in MSDB medium (*p* < 0.05). There were no significant differences between apoptosis and necrosis in cells cryopreserved derived from fragments or isolated cells.

On the other hand, among the samples of Group 2M (<30% sMitoSense+), the percentages of sDBA+ with sMitoSense+ or wMitoSense+ derived from fragments did not significantly differ among the cryopreservation media. Conversely, the percentage of necrotic cells (7AAD+) was 27.03 ± 5.80% in sDBA+ derived from frozen fragments in MSDB medium (Table 4), significantly higher than in MTDB, MTD, and MSD media (*p* < 0.05).

Moreover, after thawing of the isolated spermatogonial cells, the amounts of SSC with intact mitochondrial membranes in MSDB (34.40 ± 23%) and MSD (27.7 ± 8.70%) media were significantly different from those in MTDB and MTD freezing media (*p* < 0.05). Finally, the comparison of the percentage of sMitoSense+ cells between fragments and isolated cells belonging to Group 2M (Table 4), frozen in MSDB (12.41 ± 5.19 and 34.40 ± 23%, respectively) or MSD medium (16.64 ± 8.13 and 27.7 ± 8.70%, respectively), showed significant differences (*p* < 0.05).

### Cryopreservation of Isolated Spermatogonial Cells Before and After *in vitro* Proliferation

Other samples were analyzed by FC, to detect SSC in isolated spermatogonial cells by DBA staining as well as to evaluate mitochondrial activity in these cells. These parameters were estimated for freshly isolated spermatogonial cells, cryopreserved SSC, and cryopreserved SSC after proliferation in MSDB freezing medium. Among samples, the freshly isolated spermatogonial cells showed 7.4 ± 0.33% of SSC, a significantly higher amount than that of cryopreserved SSC (1.46 ± 0.08%; *p* < 0.05), but not significantly different than that of cryopreserved SSC after proliferation (6.29 ± 0.04; *p* > 0.05). In this case, wDBA+ cells that correspond to the SSC in early differentiation express significant differences (*p* < 0.05) with wDBA+ cells of freshly isolated spermatogonial and cryopreserved SSC after proliferation. Moreover, the percentages of cells displaying intact mitochondrial membranes, early apoptosis, necrosis, and DBA-negative (differentiated) cells did not show significance (Table 5).

**Table 5:** Mean percentage of spermatogonial stem cell before and after *in vitro* culture and freeze in MSDB freezing medium.

		$\bar{\text{X}}$ **±** **SE**					
**Samples**	**n**	**sDBA+**	**wDBA+**	**DBA-**	**sMitoSense**	**wMitoSense**	**7AAD+**
Fresh *in vitro* culture	15	6.4 ± 0.3^a^	21.4 ± 1.9^a^	69.3 ± 2.9^a^	81.3 ± 2.2^a^	8.3± 3.0^a^	8,0 ± 0.7^a^
Cryopreserved before *in vitro* culture	15	1.5 ± 0.1^b^	5.4 ± 0.1^b^	87.6 ± 2.1^a^	70.8 ± 2.9^a^	14.0 ± 1.3^a^	13,3 ± 3,2^a^
Cryopreserved after *in vitro* culture	15	6.30 ± 0.4ª	11.1 ± 2.6^a^	75.9 ± 2.8^a^	77.1 ± 2.8^a^	8.1 ± 0.7^a^	8,0 ± 2,0^a^

a,b *Different letters show statistical differences*.

## Discussion

Alpaca is an important resource for the Peruvian economy due to its high-quality meat and fiber, but its reproductive rate is low. Alpaca testes are relatively small in proportion to the body (51, 52), and high variability exists among adult animals (53). The season does not affect the testes' size but shows age-related size variation abnormalities, from 0.6 g at 6 months to 13.6 g at 36 months (54). In our case, we used the adult's testes collected all year from alpacas slaughtered at 4–6 years, and the variation of testicular weight ranged from 8 to 19.94 g, probably the testes around 8 g showed pathological conditions in alpaca. On the other hand, the male pathological condition has a high incidence in slaughtered animals at about 18.1% with an increased incidence of testicular abnormalities: hypoplasia 10.8%, cryptorchidism 3%, ectopic testes 1.9%, and cysts 14.5% (53). These pathological conditions could explain the high variation among samples (Tables 1–3) in the round spermatogonial cell and sperm concentration, low viability with trypan blue dye, and low mitochondrial activity after cryopreservation.

However, SSC isolation, molecular characterization, and cryopreservation can substantially improve the genetic conservation and reproductive fitness of these animals. In this frame, cell viability has often been evaluated by staining with trypan blue, an exclusion dye that marks only living cells (55, 56). However, this method can lead to overestimating cell viability because it cannot discriminate living cells from cells in early apoptosis. For this reason, in the present study, trypan staining was used in combination with the evaluation of mitochondrial activity by FC using the fluorochromes MitoSense and 7AAD; the first allows to determine the integrity of mitochondrial membranes while undergoing early apoptosis, and the second highlights cells necrosis. Likewise, the DBA marker was used to identify SSC.

In our assays, the highest percentage of cell viability upon trypan blue staining was observed in cells recovered from fragments of testes tissue, in medium supplemented with DMSO and sucrose or trehalose; there were statistical differences in good samples of Group 1 (Table 2) on cell derived from fragments and isolated cell.

Interestingly, freezing biopsies with DMSO results in low recovery and functionality of spermatogonia in mouse, rabbit, hamster, and monkey allotransplants or xenotransplants (39, 57), whereas in pig and human post-thawed biopsies, spermatogenesis can be completed (11, 58).

Notably, our results have demonstrated the possibility of maintaining frozen alpaca SSC for long periods (1–166 days), similar to mice (59). This opens the possibility of storing frozen alpaca samples for several years, as also observed in mice (60), if collected within 24 h from death of the alpaca.

In our work, isolated cells and testicular biopsies showed different cryopreservation efficiencies and different requirements for CPA. However, previous reports have shown variable results concerning the type of tissue being frozen. For example, Pacchiarotti et al. (61) found no differences between the efficiency of freezing between isolated cell suspensions and tissues, whereas Yango et al. (62) showed that the viability of cell suspensions was greater than that of biopsies in adults, but not in newborns, thus suggesting that age is an important factor for the selection of the right method for the cryopreservation of testicular tissue or isolated cells. In our study, only testicular samples from adult alpacas were used; therefore, the differences in post-thaw cell viability between biopsies and cell suspensions are consistent with the aforementioned studies. However, in mice, freezing of testicular tissue was more effective than freezing of cell suspensions (63). This report contrasts with our results, since we observed better mitochondrial activity in thawed isolated alpaca cells.

In our experiment with FC using MitoSense, the most prominent and significant statistical differences in the cryopreserved isolated cells occurred in the MSDB medium. In particular, the highest mitochondrial activity was observed in isolated cells in freezing medium supplemented with DMSO, sucrose, and black maca. Cells from testicular biopsies also showed good results about the conservation of mitochondrial activity when frozen in medium supplemented with DMSO, trehalose, and black maca but not significant. However, it is the way to easy conservation of testes tissue, cheaper, and without complex methods. The detected differences between frozen fragments and isolated cells may be due to the differing physical dimensions of the samples, the distribution of cryoprotectants, and the uniformity of temperature during cooling (63–65). In mice, better results have been observed when freezing whole testes (66), whereas in rats, freezing of small testicular biopsies showed better results than those obtained with fragments twice the size (67). On the other hand, the size of the testicular biopsies did not significantly affect the efficiency of cryopreservation either in pigs (58) or in prepubertal children (9, 38, 68–70). The biopsy size in our previous study was 8 × 2 × 2 mm (26); therefore, in this work, we used the same size and observed an average viability of 57.2% in samples of good quality (>50% viability), suggesting that this size is adequate for freezing of alpaca testicular biopsies. On the other hand, the size of the biopsies used for human SSC cryopreservation was not indicated. However, the freezing of testicular biopsies is recommended (71).

Interestingly, we found that media supplemented with non-permeable CPA, such as sucrose, exerted a greater cryoprotective effect on isolated cells. This sugar has previously been shown to mediate better results than trehalose when freezing bovine spermatozoa (72). In the case of pigs, it has been observed that sucrose at 280 mM allowed better vitality of cryopreserved SSC (29), which is consistent with our results for freezing of isolated alpaca SSC. On the other hand, Jung et al. (73) found that stabilization of isolated mouse spermatogonia with trehalose reduced the cytotoxicity of the cells and improved post-thaw survival. Similar results were found by Syvyk et al. (74) in isolated rat SSC. Sucrose was not used in these studies; however, the results of these studies differ from ours, since we observed that trehalose did not improve mitochondrial activity in isolated cells. This could indicate that the efficacy of CPA may be species dependent. On the other hand, previous research in tissue freezing indicates that the use of trehalose confers greater cellular vitality (75), consistently with our results, as trehalose was better than sucrose for freezing of testicular tissue.

Moreover, antioxidants can improve the cryopreservation process (13, 76, 77), and maca is considered a natural antioxidant because it can help maintain DNA integrity. In particular, some derivatives of maca can protect cells against oxidative stress (31, 33), due to the presence of phytochemicals capable of scavenging free radicals, and thus can help fight chronic inflammation (33). In the current study, we observed that the presence of black maca helps alpaca isolated SSC to retain efficient mitochondrial activity, suggesting the presence of maca metabolites with antioxidant properties that protect the mitochondria as observed in isolated cell of good (>30% sMitoSense+) and regular (<30% sMitoSense+) quality of samples (Tables 3, 4). In addition to the antioxidant activity of maca, high concentrations of sucrose may play a key role in cryoprotection (45, 47, 49), since, as previously stated, the presence of sucrose as a cryoprotectant improves post-thaw cell viability (MSDB medium). Therefore, antioxidants and cryoprotectant components of maca can be useful for cryopreservation; this phenomenon could be explained by the fact that maca belongs to the Brassicaceae family, which easily adapts to heat and hydric stress conditions at the heights of the Andes (78). The phytochemicals of maca allow its survival in the difficult conditions of its habitat, and this could explain their protective effect in cryopreservation.

In the present study, DBA staining, together with the FlowCellect MitoPotential Red kit, allowed to identify intact mitochondrial membrane activity (sMitoSense+), early apoptosis (wMitoSense+), and necrosis (7AAD+) in SSC. This analysis, carried out by FC, makes it possible to better recognize the events and damages generated by the cryopreservation process and the degree of effective conservation through this process, compensating the insufficient indications provided by trypan blue staining alone.

In conclusion, our study showed that the supplementation of natural atomized black maca in the cryopreservation medium improves survival and preserves intact mitochondrial membrane activity in alpaca SSC. Furthermore, another CPA, trehalose, was found to be a better cryoprotectant for testicular fragments, whereas sucrose would be more effective for cryopreserving isolated SSC. We conclude that it is possible to cryopreserve alpaca SSC collected after 24 h from death in the form of biopsies or isolated cells in the presence of DMSO and natural supplement of black maca in the freezing media for cryopreserved good and regular quality samples. Furthermore, we recommend the use of these MSDB media for the cryopreservation of isolated SSC after *in vitro* proliferation.

## Data Availability Statement

The raw data supporting the conclusions of this article will be made available by the authors, without undue reservation.

## Ethics Statement

The animal study was reviewed and approved by Comité Institucional de ética para el uso de animales CIEA, Universidad Peruana Cayetano Heredia Codigo SIDISI N° 0000066755.

## Author Contributions

MV did cryopreservation protocols for isolated and testis tissue and analysis by CF. JR has done SSC *in vitro* proliferation. ZB did statistical analyses and discussion. GG, MV, and JR did the analysis of the results. All authors contributed to the article and approved the submitted version.

## Conflict of Interest

The authors declare that the research was conducted in the absence of any commercial or financial relationships that could be construed as a potential conflict of interest.
